# National Clinical Database feedback implementation for quality improvement of cancer treatment in Japan: from good to great through transparency

**DOI:** 10.1007/s00595-015-1146-y

**Published:** 2015-03-24

**Authors:** Mitsukazu Gotoh, Hiroaki Miyata, Hideki Hashimoto, Go Wakabayashi, Hiroyuki Konno, Shuichi Miyakawa, Kenichi Sugihara, Masaki Mori, Susumu Satomi, Norihiro Kokudo, Tadashi Iwanaka

**Affiliations:** 1 National Clinical Database, 1-8-3 Marunouchi, Chiyoda-ku, Tokyo, Japan; 2 The Japanese Society of Gastroenterological Surgery (JSGS), Database Committee, 1-14-1-501 Shintomi, Chuo-ku, Tokyo, 104-0041 Japan; 3 Board Certification Committee of JSGS, Tokyo, Japan

**Keywords:** Gastrointestinal surgery, National Clinical Database, Nationwide web-based database, Mortality, Risk model

## Abstract

The National Clinical Database (NCD) of Japan was established in April, 2010 with ten surgical subspecialty societies on the platform of the Japan Surgical Society. Registrations began in 2011 and over 4,000,000 cases from more than 4100 facilities were registered over a 3-year period. The gastroenterological section of the NCD collaborates with the American College of Surgeons’ National Surgical Quality Improvement Program, which shares a similar goal of developing a standardized surgical database for surgical quality improvement, with similar variables for risk adjustment. Risk models of mortality for eight procedures; namely, esophagectomy, partial/total gastrectomy, right hemicolectomy, low anterior resection, hepatectomy, pancreaticoduodenectomy, and surgery for acute diffuse peritonitis, have been established, and feedback reports to participants will be implemented. The outcome measures of this study were 30-day mortality and operative mortality. In this review, we examine the eight risk models, compare the procedural outcomes, outline the feedback reporting, and discuss the future evolution of the NCD.

## Introduction

Until recently, no nationwide data on cancer were available in the field of gastroenterological surgery in Japan. In 2006, the Japanese Society of Gastroenterological Surgery (JSGS) formed a committee to devise a database to track surgical patients treated in Japan over the 3 years from 2006 to 2008, and reported relatively low mortality rates for the major surgical procedures [1, 2]. The JSGS acknowledged the importance of risk-adjusted surgical outcomes for accurate comparisons and quality improvement; thus, in April, 2010, it created the database as a subset of the National Clinical Database (NCD) of Japan with major support from the Japan Surgical Society (JSS). Eight other surgical professional societies, including the Japanese Society for Cardiovascular Surgery, the Japanese Society for Vascular Surgery, the Japanese Association for Thoracic Surgery, the Japanese Association for Chest Surgery, the Japanese Society of Pediatric Surgeons, the Japanese Breast Cancer Society, the Japan Association of Endocrine Surgeons, and the Japanese Society of Thyroid Surgery, joined the NCD. Registrations began in 2011, since when more than 4100 facilities have enrolled and over 4,000,000 cases have been registered over a 3-year period.

The gastroenterological section of the NCD collaborates with the American College of Surgeons National Surgical Quality Improvement Program (ACS-NSQIP) [3], which shares a similar goal of developing a standardized surgical database for quality improvement. The NSQIP was originally developed in the 1990s by the United States Veterans’ Health Administration and led to marked improvement in surgical quality [4]. The American College of Surgeons (ACS) initiated the ACS-NSQIP in 2006 and demonstrated improved surgical outcomes across all participating hospitals in the private sector [5]. The core members of the NCD joined the meetings and seminars of the ACS-NSQIP and debated various aspects of clinical databases, such as data collection methods and public relations [3]. In addition, the NCD implemented the same items as those of the ACS-NSQIP to conduct international cooperative studies. Reliable 30-day outcomes, including mortality and morbidity, serve as a quality improvement catalyst for ACS-NSQIP-participating institutions. Risk adjustment is a key component of the ACS-NSQIP and most variables included in risk adjustment models focus on patient factors and comorbidities. In this article, we focused on the gastrointestinal surgery subset of the NCD. All cases are input with items representing the surgical performance in each specialty for the following eight procedures: esophagectomy (Eso), total/distal gastrectomy (TG/DG), right hemicolectomy (RHC), low anterior resection (LAR), hepatectomy performed for more than one segment apart from the lateral segment (Hx), pancreaticoduodenectomy (PD), and surgery for acute diffuse peritonitis (ADP). Risk models of mortality for each procedure were created using approximately 120,000 cases registered in 2011, and each model has been accepted and published in peer-reviewed journals [6–13]. We review the results and discuss the future evolution of the NCD using these risk models in terms of the surgical quality improvement program in Japan.

## NCD data entry system

Submitting cases to the NCD is a prerequisite for all member institutions of the JSS and JSGS, and only registered cases can be used for board certification [3]. To assure the traceability of data, the NCD continuously tracks persons who approve data, persons in departments who are in charge of annual cases, and persons responsible for data entry, through its web-based data management system. The NCD also continuously validates data consistency through random site visits.

The NCD variables are almost identical to those applied in the ACS-NSQIP (http://www.site.acsnsqip.org/wp-content/uploads/2013/10/ACSNSQIP.PUF_.UserGuide.2012.pdf#search=‘user+guide+for+the+2012+ACS+NSQIP). The potential independent variables include patient demographics, pre-existing comorbidities, preoperative laboratory values, and perioperative data. The demographic variables include age, sex, smoking status, and drinking status. Patients were categorized according to whether they were brought to hospital directly, by ambulance. General factors such as the patient’s body mass index (BMI) and preoperative functional status, defined as independent, partially dependent, or totally dependent, according to their ability to perform activities of daily living (ADL) in the 30 days prior to surgery and immediately before surgery, were also considered. We evaluated the physical status classification by the American Society of Anesthesiologists (ASA) and considered pre-existing comorbidities, including the cardiovascular status, respiratory status, renal status, hematological status, oncological status, preoperative blood transfusion, chronic steroid use, ascites, sepsis, diabetes, open wound, and pregnancy. The laboratory parameters included in the analysis were the white blood cell count, hemoglobin level, hematocrit, platelet count, prothrombin time, and activated partial thromboplastin time, as well as the serum levels of albumin, total bilirubin, aspartate amino transferase, alanine aminotransferase, alkaline phosphatase, urea nitrogen, creatinine, sodium, hemoglobin A1c, and C-reactive protein. The length of surgery, intraoperative blood loss, amount of transfusion, and any accident during the operation were also considered.

Postoperative outcomes evaluated 30 days after surgery were categorized according to the Clavien and Dindo classification [14]. The outcomes included relaparotomy within 30 days after surgery, wound events, anastomotic leak, respiratory events, urinary tract events, central nervous system events, cardiac events, other events, systemic sepsis, sepsis, systemic inflammatory response syndrome, and 24 other complications added by the NCD. For Hx procedures, the indications for surgery and resected subsegments (S1–S8) were included as preoperative variables to create risk models [9].

### Outcome measures and statistical analysis

The outcome measures of this study were 30-day mortality and operative mortality. The former was defined as death within 30 days of surgery, regardless of the patient’s geographical location, even if the patient had been discharged from hospital. The latter was defined as death within the index hospitalization period, regardless of the length of hospital stay (up to 90 days), as well as any death after discharge, up to 30 days after surgery. Data were randomly assigned into two subsets that were split 80/20: the first, for model development, and the second, for validation. The two sets of logistic models (30-day mortality and operative mortality) were constructed for dataset development using step-wise selection of the predictors with a probability (*p*) value for inclusion of 0.05. A “goodness-of-fit” test was performed to assess how well the model could discriminate between patient survival and death. The receiver operating characteristic (ROC) curves for the 30-day and operative mortalities were created for the validation dataset. An ROC curve is a plot of a test’s true-positive rate (sensitivity) versus its false-positive rate (1—specificity). Model calibration, being the degree to which the observed outcomes matched the predicted outcomes from the model across a group of patients, was examined by comparing the observed and predicted averages with each of 10 equally sized subgroups, arranged in the order of increasing patient risk.

### Case number and participating hospitals for each procedure and mortality rates

The NCD is a nationwide project in cooperation with Japan’s board certification system in surgery, for which more than 1,200,000 surgical cases from over 3500 hospitals were collected in 2011. The number of participating hospitals in the gastroenterological section was 2045 at the time of the analysis (July, 2012). Among these cases, approximately 120,000 were used to create the risk models. Table 1 lists the number of cases for each procedure and the number of hospitals performing the respective procedure with its ratio to the total number of hospitals (%). Most procedures, except for ADP, were performed for cancer. Emergency surgery was most common for ADP (93 %). The 30-day mortality and operative mortality rates for the eight procedures were as follows: Eso, 1.2/3.4; TG, 0.9/2.3; DG, 0.5/1.2; RHC, 1.1/2.3; LAR, 0.4/0.9; HX, 2.0/4.0; PD, 1.2/2.8; and ADP, 9.0/14.1 %, respectively (Table 1). The operative mortality for each procedure, apart from ADP, was more than twice that of the 30-day mortality.

**Table 1:** Registered cases used to create risk models for 8 surgical procedures [6–13]

	Eso	TG	DG	RHC	LAR	Hx	PD	ADP
Registered cases	5354	20,011	33,917	19,070	16,695	7732	8575	8482
Participating hospitals	713	1623	1737	1689	1620	987	1167	1285
(%)	34.9	79.4	84.9	82.6	79.2	48.3	57.1	62.8
30-day mortality (%)	1.2	0.9	0.5	1.1	0.4	2.0	1.2	9.0
Operative mortality (%)	3.4	2.3	1.2	2.3	0.9	4.0	2.8	14.1
Cancer surgery (%)	98.4	98.5	99.9	92.6	98.5	94.5	91.4	10.8
Emergent case (%)	0.8	2.0	0.9	8.4	1.1	0.8	0.9	92.9

Esophagectomy (Eso), total/distal gastrectomy (TG/DG), right hemicolectomy (RHC), low anterior resection (LAR), hepatectomy performed for >1 segment except for the lateral segment (Hx), pancreaticoduodenectomy (PD), and operation for acute diffuse peritonitis (ADP)

### Risk models in the eight procedures

The 30-day mortality and operative mortality risk models for the eight procedures were created, and the C-index for those in the validation data sets was as follows: Eso, 0.767/0.742; TG, 0.811/0.824; DG, 0.785/0.798; RHC, 0.836/0.854; LAR, 0.75/0.766; HX, 0.714/0.761; PD, 0.675/0.725; and ADP, 0.851/0.852, respectively (Tables 2, 3). The final logistic models for the 30-day mortality with odds ratios for the eight procedures are listed in Table 2. Age; sex; emergency surgery; ADL; ASA class; BMI; cardiovascular, pulmonary, and renal comorbidities; and other patient conditions such as disseminated cancer, ascites, preoperative transfusion, bleeding disorder, diabetes, weight loss, sepsis, and chronic steroid use, including 121 variables, were found to be risk factors for certain procedures. Age, ADL ASA, BMI, disseminated cancer, bleeding disorder, and weight loss appeared to be common risk factors in most of the procedures. Table 3 lists the final logistic models for the operative mortality with odds ratios for the eight procedures, including 159 variables. New and additional 38 variables were captured for these models.

**Table 2:** Risk models for 30-day mortality after 8 gastrointestinal procedures (refs 6–13)

Variables	Eso	TG	DG	RHC	LAR	Hx	PD	ADP
Age category	1.5	1.2	1.2		1.3	1.4	1.3	1.2
Male sex						1.6	2.0	
Ambulance transport								1.4
Emergent surgery				1.9		3.8	4.3	
ADL within 30 days before surgery								
Any assistance	4.2					2.1		
Total			3.0					
ADL immediately before surgery								
Any assistance		2.1		2.8				
Total								1.4
ASA								
Class 3				2.3				2.7
Class 4								4.3
Class 5								8.7
Class 3, 4, 5			2.0			2.0	2.2	
Class 4, 5		9.4		4.0				
BMI								
>25 kg/m^2^							2.4	
>30 kg/m^2^					7.0			
Congestive heart failure				2.3				
Previous cardiac surgery		2.3						
Myocardial infarction			3.1					
Previous PCI								2.0
Previous PVD surgery					6.2			2.5
Cerebrovascular disease			2.1					
COPD							2.4	
Preoperative pneumonia			2.8					
Respiratory distress								1.6
Acute renal failure				3.2				
Preoperative dialysis		3.9						
Cancer with multiple metastases				2.2				
Disseminated cancer		2.6			4.9			2.2
Preoperative transfusion		1.9			5.4			1.6
Bleeding disorder without treatment			3.2		5.2			1.6
Bleeding disorder							4.4	
Diabetes		2.2						
Smoking within 1 year	2.6							
Ascites		2.0				2.1		
Without control			3.0					
Chronic steroid use								1.7
Weight loss	2.4		2.3					
Sepsis				2.0				
Habitual alcohol consumption			1.6					
WBC								
>12,000/μl	3.7		3.7					
>9000/μl				1.5				
<4000/μl	2.8							1.4
Hemoglobin								
M < 13.5 g/dl, F < 12.5 g/dl		1.7	1.8					
<10.0 g/dl								1.3
Platelet								
>400,000/μl	2.5							
<150,000/μl								1.5
<120,000/μl				1.9	5.0	1.7		
<80,000/μl		3.1						1.5
<50,000/μl				5.6				
Albumin								
<4.0 g/dl				2.0	3.4			
<3.5 g/dl		1.7	1.5			2.0		
<2.0 g/dl								1.7
Total bilirubin								
>3.0 mg/dl				3.1				1.7
>2.0 mg/dl		2.9						
AST								
>35 U/l		2.3		3.1		2.3		1.4
ALP								
>600 U/l		2.5						1.7
>340 U/l		1.7	2.2					
BUN								
>25 mg/dl		1.9			2.5			1.4
>20 mg/dl								1.8
<8.0 mg/dl							2.3	
Creatinine								
>2.0 mg/dl						3.9		
>1.2 mg/dl			1.8					
Serum Na								
>145 mEq/l								1.7
<138 mEq/l				2.1	3.6			
<135 mEq/l	3.6		2.5					
<130 mEq/l								1.7
CRP								
<10.0 mg/dl								1.5
APTT								
>40 s							3.2	
PT-INR								
>1.25		2.2	2.0					
>1.1	2.0			1.5		1.7		
Non-tumor bearing								0.6
Surgical procedures						#1		
Indication for surgery						#2		

#1 Hepatectomy with S8 (2.2), hepatectomy with revascularization (3.8)

#2 Hilar bile duct carcinoma (2.5), gallbladder cancer (4.1)

*ADL*, Activities of daily living, PT-INR Prothrombin time-international normalized ratio, *WBC* white blood cells, *ASA* American society of anesthesiologists, *ADL* activities of daily living, *PCI* percutaneous coronary intervention, *COPD* chronic obstructive pulmonary disease, *AST* aspartate amino transferase, *ALP* alkaline phosphatase, *APTT* activated partial thromboplastin time

**Table 3:** Risk models for operative mortality after 8 gastrointestinal procedures [6–13]

Variables	Eso	TG	DG	RHC	LAR	Hx	PD	ADP
Age category	1.4	1.3	1.3	1.1	1.4	1.4	1.3	1.3
Male sex	2.3				1.9	1.5		
Emergent surgery		1.7	1.9	1.9		2.8		
ADL within 30 days before surgery								
Any assistance	4.7					2.8	2.5	
Total								1.6
ADL immediately before surgery								
Any assistance		2.0		2.5	2.5			1.4
Total			3.0		2.9			
ASA								
Class 3		1.8		1.6				2.3
Class 4								4.7
Class 5								6.5
Class 3, 4, 5			1.9			2.0	2.1	
Class 4, 5		5.2		2.9				
BMI								
>25 kg/m^2^							1.9	
>30 kg/m^2^					4.6			
Congestive heart failure				2.2				
Angina							2.6	
Previous PVD surgery				3.1	5.8			
Cerebrovascular disease			1.8					
Cerebrovascular accident		1.9						
Respiratory distress								
Any		1.7	2.4		2.9		2.4	
COPD	2.1					2.0		
Preoperative pneumonia						3.8		1.4
Preoperative dialysis		2.6		2.1				
Cancer metastasis/relapse	4.5			1.6				
Disseminated cancer		3.5	2.9	3.1	2.8			2.1
Preoperative transfusion					2.6			1.8
Bleeding disorder without therapy								1.6
Brinkman index							1.6	
Ascites								
Any		1.8		1.6	4.0	1.9		
Without control			2.8					
Chronic steroid use			2.8	2.0				1.9
Weight loss	2.0	1.6	2.2	1.6			2.1	1.4
Sepsis				1.7				
WBC								
>11,000/μl		2.0	2.5				3.1	
>9000/μl				1.6				
<4500/μl	1.8							1.5
<3500/μl		1.6						
Hemoglobin								
M < 13.5 g/dl, F < 12.5 g/dl					2.6			1.3
<10 g/dl						1.8		
Hematocrit								
M > 48 %, F > 42 %					3.6			
M < 37 %, F < 32 %			1.4	1.4				
<30 %		1.3						1.2
Platelet								
<120,000/μl	2.0		2.0	1.7	3.4	1.6	2.1	1.4
<80,000/μl				2.6		2.1		
Albumin								
<3.8 g/dl			1.7					
<3.5 g/dl	2.2	1.4				1.6		
<3.0 g/dl		1.4		1.5		1.7		1.4
<2.5 g/dl					2.7			
<2.0 g/dl								1.5
Total bilirubin								
>3.0 mg/dl								2.0
>2.0 mg/dl		2.8	2.6					
>1.0 mg/dl				1.6				
AST								
>40 U/l			1.5	2.7	1.9	1.7		
>35 U/l		1.7						1.4
ALP								
>600 U/l		3.1						1.6
>340 U/l			1.6					
BUN								
>60 mg/dl				2.4				
>25 mg/dl								1.3
>20 mg/dl								1.8
<8 mg/dl	2.6			1.6				
Creatinine								
>2.0 mg/dl								1.5
>1.2 mg/dl			1.8					
Serum Na								
>145 mEq/l				1.9				
<138 mEq/l	2.1	1.4		1.9	2.5			
<135 mEq/l			2.3					
<130 mEq/l								1.8
CRP								
<10.0 mg/dl								1.5
APTT								
>40 s			1.6				2.0	
PT-INR								
>1.25	3.0	1.9						
>1.1			1.5	1.4		1.4	1.5	
Non-tumor bearing								0.5
Surgical procedure		#1				#2		
indication for surgery						#3		

#1 Pancreatico splenectomy (2.2)

#2 Hepatectomy with S1 (1.6), S7 (1.6), S8 (2.0), left tri-segmentectomy with S1 resection (3.9), hepatectomy with revascularization (3.0)

#3 Intrahepatic cholangiocarcinoma (1.8), hilar bile duct carcinoma (2.0), gallbladder cancer (3.2)

### Feedback implementation (risk calculator)

A risk-adjusted analysis based on nationwide data allows personnel to establish and provide feedback on the risks that patients face before undergoing a procedure. On the basis of these objective data, healthcare professionals can then determine the treatment indicators and obtain informed consent. The risk calculator for all eight procedures will be available soon, on the websites of the hospitals that are a part of NCD, although the calculators for TG, PD, Hx, Eso, RHC, and LAR are currently available (February, 2015). The real-time feedback system gives the predicted mortality of patients simultaneously with data input. Standardized information on patient risk and predicted mortality can be reformulated as case reports and shared at conferences.

**Fig. 1:**
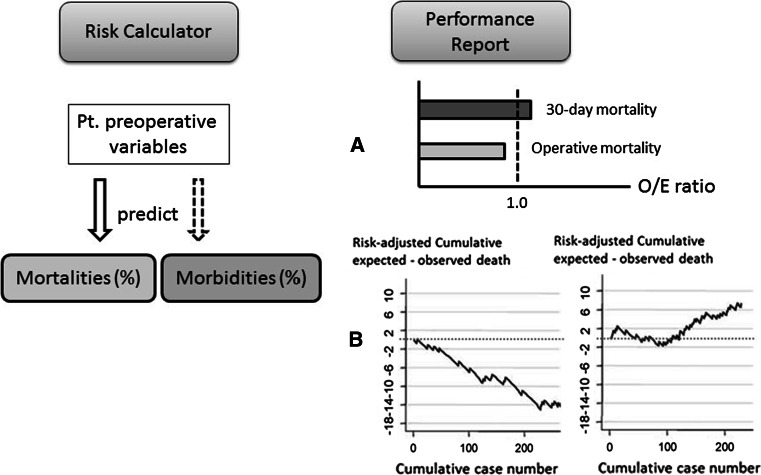
The National Cancer Database feedback system includes a risk calculator for the mortality and morbidity of preoperative patients (*left schema*) and performance reports of each participating hospital (*right schema*). The latter includes each facility’s severity-adjusted clinical performance (*benchmark*) in comparison with the national data (**a**) and the risk-adjusted cumulative expected–observed death (**b**). Better (*right*) or worse (*left*) outcomes can be detected by the monitoring report

The NCD will soon be able to provide data on each facility’s severity-adjusted clinical performance (benchmark), which can be compared with national data (Fig. 1a). Cumulative observed–expected mortality can be traced periodically after each operation and used to detect special cause variations showing better (right) and worse (left) outcomes (Fig. 1b).

## Future evolution of NCD

### A complete data acquisition system link to board certification

More than 4,000,000 cases were retrieved from the NCD during the 3 years before April 2013. The number of esophagectomy and pneumonectomy cases registered in the NCD accounted for approximately 95 % of all cases registered in the Regional Bureau of Health and Welfare. Thus, most cases in Japan appear to be captured by the NCD system. This NCD project started with support from Health and Labor Sciences Research Grants by the Ministry of Health Labour and Welfare (Principal Investigators; MG, T.I.) and considerable funding from the JSGS and JSS. Participating institutions can now use the database system at no cost; however, it is mandatory for the institutions to participate in the benchmarking project when applying for the board certification system. Currently, the board certification system is operating adequately on the web for surgical society members and allows members to obtain information on their cases being used to assess a member’s qualifications for certification during a certain period. Any applicant who has a sufficient number of cases for application no longer needs to write case reports. All participating healthcare professionals use information acquired from the NCD. Moreover, the board certification system itself can be revalidated using the surgical improvement program of the NCD.

### Share benefits and costs of the NCD with relevant stakeholders

A previous study by Hall et al. [5] showed that participation in the benchmark reporting system of the ACS-NSQIP improved surgical outcomes across all participating hospitals in the private sector. Improvement is reflected for both poor- and well-performing facilities. They speculated in the model using 183 participating hospitals that each institution may have avoided 200–500 complications and 12–36 deaths. Participation in the ACS-NSQIP benefits patients, surgeons, and hospitals and costs 10,000–29,000 (US$) depending on the ACS-NSQIP options [15.^]^

In the gastroenterological section, risk models of mortality for the eight procedures were created to enable feedback. Simultaneously, risk models of morbidities for the eight procedures are being created to enable feedback for the next year. Currently, the database system is built up to enable efficient provision of benchmark reports to each institute. The benefits and costs can now be shared with the relevant stakeholders. A participation fee depending on the number of cases for retrieval is expected to be charged by the NCD to each hospital. Research grants from various sources are also expected to support clinical investigations using the NCD data.

### Eliminating burden on physicians and maintaining data accuracy

To avoid burdening physicians, the NCD allows data entry by other medical staff members. The NCD data entry privileges allow people other than physicians to enter the data. An appropriate educational system for data managers would be mandatory to maintain the accuracy of data and reduce the burden on physicians. This could be achieved by holding an annual data manager educational meeting and eventually introducing an e-learning system. The JSGS is planning to create an audit committee separately from the NCD, with the goal of achieving accurate data inputs and of educating data managers.

### Quality improvement of surgical care for cancer patients

The NCD generalizes site-specific cancer registries by taking advantage of their excellent organizing ability. Some site-specific cancer registries have already been combined with the NCD [16]. Cooperation between the NCD and site-specific cancer registries can establish a valuable platform upon which a cancer care plan can be developed in Japan. Furthermore, information on the prognosis of cancer patients gathered using population- and hospital-based cancer registries can enable efficient data accumulation into the NCD.

Currently, quality assessment of hospitals is being carried out using the Diagnosis Procedure Combination (DPC) data from the participating hospitals [17, 18]. The DPC data include variables for preoperative morbidities, cancer variables, and postoperative complications, but they are based mainly on administrative claim data. A low participation rate by very small hospitals in the DPC system covers 50% of institutions conducting surgical services [17] and hampers complete enumeration. The NCD is a quality assessment and improvement program in which clinical data are used with a high collection rate (95 %). Site-specific cancer registries in the NCD would not only be more accurate and suitable for perioperative assessment, but also for long-term outcomes of cancer patients.

### Further improvements through transparency

Public reporting and transparency are being demanded by multiple stakeholders [19, 20]. Although it has been shown that performance data released to the public promote quality improvement activity at the hospital level [21, 22], opponents counter that public reporting induces gaming and other unintended consequences such as “cherry picking” (hospitals selecting lower-risk patients to avoid poorer outcomes) or losing patients to better-performing hospitals [23]. With the consent of participating surgical societies, the NCD stated that the performance of each institute would be fed back only to respective institutes but not to the general public. This practice is similar to that of the ACS-NSQIP, from which a report is prepared for administrators and surgical services staff to compare their risk-adjusted surgical outcomes with those of participating sites that are blinded to data other than their own.

In 2012, the ACS-NSQIP partnered with the Centers for Medicare and Medicaid Services (CMS) to promote public reporting and transparency of surgical outcomes [24]. Although there were few measurable differences between CMS-NSQIP-participating and CMS-NSQIP-nonparticipating hospitals, it was found that of all possible hospital structural characteristics, only the teaching hospital status predicted participation in the CMS-NSQIP public reporting initiative. It may be a challenge for participating hospitals to show their performance to the general public. There is an interesting study by Sherman et al. [25, who investigated surgeons’ perceptions of public reporting of hospital and individual surgeon quality. They stated that surgeons recommended patient education, simplified data presentation, and continued risk-adjustment refinement, and conducted an internal review before public reporting to make public reporting more acceptable for them. Linkage between hospital information systems and the NCD registry system may improve data accuracy and save costs. Presentation of care quality is increasingly regarded as imperative to support patients’ choice and efficiency of care provision. We want medical professionals to realize that good to great performance can be achieved only through transparency for providers and patients.

## Abbreviations

NCD: National Clinical Database
ACS NSQIP: The American College of Surgeons National Surgical Quality Improvement Program
ASA: American Society of Anesthesiologists
CNS: Central nervous system
COPD: Chronic obstructive pulmonary disease
DIC: Disseminated intravascular coagulation
JSS: The Japan Surgical Society
JSGS: The Japanese Society of Gastroenterological Surgery
ROC: Receiver operating characteristic
SIRS: Systemic inflammatory response syndrome
SSI: Surgical site infection
